# Bright Thermo-resilient and Promiscuous Zombie Protein for Lighting Applications

**DOI:** 10.1021/acsmaterialslett.5c00653

**Published:** 2025-07-25

**Authors:** Marta Patrian, Marco Hasler, Jesús A. Banda-Vázquez, Evgenia Borisova, Juan Pablo Fuenzalida Werner, Rubén D. Costa

**Affiliations:** 9184 Technical University of Munich, Campus Straubing for Sustainability and Biotechnology, Chair of Biogenic Functional Materials, Schulgasse, 22, Straubing 94315, Germany

## Abstract

Proteins are at the forefront of materials science, with implementations in optical, electrical, and structural materials for transformative and sustainable technologies. Within the biohybrid light-emitting diode (BioHLED) concept, replacing toxic and/or rare photon filters with classical β-barrel fluorescent proteins (FPs) that must withstand irradiation, temperature, oxidation, and dehydration stress, the question if FPs from extremophiles and/or living fossils might be better for lighting applications arises. We addressed this by introducing a thermostable prokaryotic FP, whose inherent promiscuity enables the design of tunable emitting proteins. Three milestones were reached: (*i*) a comprehensive phylogeny of phycobiliproteins from a large data set (182 proteins from 29 thermophiles) to identify the most versatile zombie-like phycobiliprotein (highly ancestral character), (*ii*) heterologous expression of this phycobiliprotein (SPritZ) in *Escherichia coli* and further enhancement via rational mutagenesis into a brighter and more thermal-resilient variant (eSPritZ), and (*iii*) 2.5-fold stable BioHLEDs comparing SPritZ vs eSPritZ in hydroxypropyl cellulose coatings.

## Graphical abstract

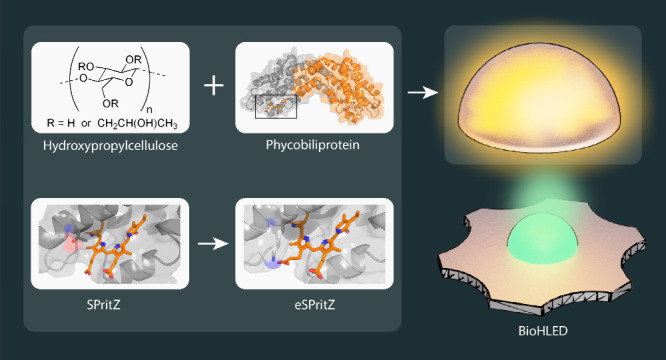

Driven by advancements in synthetic biology and protein engineering, proteins and enzymes are at the forefront of sustainable material engineering, including noninvasive imaging,^1^ regenerative 3D scaffolds,^2^ optoelectronics,^3,4^ living materials,^5,6^ and photonics.^7^ In this context, biohybrid light-emitting diodes (BioHLED) implementing fluorescent proteins (FPs) promise to substitute color down-converting filters based on rare-earth inorganic phosphors (IPs).^8–16^ This is even more critical as IPs lack efficient recycling protocols and severely contribute to ecosystem disruption due to their mining and disposal.^17a^ In contrast, protein-based color filters are sustainable and can be recycled in ways that fulfill the circular economy.^17b^

Among the known families of FPs, the classical β-barrel FPs have been widely studied for application in BioHLEDs, due to their high photoluminescence quantum yield (ϕ) and cheap and efficient recombinant production in bacteria. Here, BioHLEDs operating at high excitation powers still feature stabilities of a few hours due to (*i*) photoinduced heat generation resulting in denaturing temperatures of up to 70 °C and (*ii*) irreversible photoisomerization, H-transfer, and/or oxygen-mediated deactivation processes.^8–16^ Several strategies have been proposed to stabilize FPs for lighting spanning from (*i*) fabricating waterless coatings,^9,11,13^ to (*ii*) water-free FP isolation using sol–gel and metal organic framework (MOF) chemistry tools,^11,12,16^ to (*iii*) genetically engineered self-assembled FPs,^13,14^ and to (*iv*) FP-based cocrystals,^15^ among others.

Since β-barrel FPs have generally evolved in mesophilic marine organisms and have been traditionally optimized to serve as genetic reporters in mammalian cells, they lack an intrinsic capacity to withstand extreme conditions (i.e., temperature and irradiation stress as well as foreign environments) commonly found in technological applications. However, Nature has taught us about a myriad of organisms living in extreme conditions (i.e., extremophiles fully adapted to extreme temperatures, radiations, salinities, pH, etc.) and/or living fossils (i.e., species-poor lineages that phenotypically resemble related ancestors) dating back billions of years.^18,19^ Among them, cyanobacteria are considered the oldest living fossils, emerging 3.5 billion years ago.^20^ They and others, such as red algae and cryptomonads, share a family of light-harvesting protein systems based on phycobiliproteins (PBPs). These thermo-resilient FPs of prokaryotic origin can be proposed to replace classical β-barrel FPs in BioHLEDs.^21^ However, the limitation of this concept is twofold. On one hand, only a few chromophorylated PBPs have been produced in metabolically engineered *Escherichia coli* (*E. coli*), as they possess complicated multistep chromophorylation processes.^22^ On the other hand, mesophilic-derived PBPs, such as smURFP, have been optimized, reaching only moderate ϕ at the low-energy part of the visible spectrum, but good stabilities in BioHLEDs.^23^

Here, we expand upon these findings with the goal of identifying and redesigning ancestral-like PBPs that exhibit broad promiscuity toward bound chromophores, enabling the tuning of the emission features, while maintaining robust tolerance to harsh conditions, such as high temperatures.^24–28^ Thus, in order to find the closest living protein fossil, we first estimated the phylogeny of PBPs out of a large data set (182) from 29 known thermophilic cyanobacteria. Instead of computationally estimating the last common ancestral-PBP (as it was from an already extinct bacterium),^29^ we focused on the closest zombie-like PBP (i.e., high ancestral character present in a living organism) that is a likely direct descendant of the ancestral-PBP. This phycobiliprotein was produced in *E. coli*, showing the desired chromophore promiscuity for the native deep-red phycocyanobilin (PCB) and the orange phycoerythrobilin (PEB) chromophores, but not for phycourobilin (PUB). Among them, the orange variant featured a remarkable stability with respect to its ϕ (<10% loss) after thermal challenge at 90 °C, being of high interest for BioHLED purposes. Thus, we coined this PBP SPritZ as an abbreviation of Synechococcus phycobiliprotein thermo-resilient zombie. As a next step, we carried out a rationally designed targeted mutagenesis (T80K) to efficiently host the non-native PEB chromophore, achieving a brighter (ϕ = 53%) and more thermally robust variant (i.e., enhanced SPritZ, or eSPritZ). What is more, both SPritZ and eSPritZ fairly kept the thermal and photoluminescent features in hydroxypropyl cellulose coatings, while the eSPritZ led to BioHLEDs with a 2.5-fold enhanced stability compared to those with SPritZ.

Overall, this work highlights the potential of the PBP family, which could become another class of protein emitters for lighting purposes, with eSPritZ as the first representative.

To find the PBP living fossil, we first built up a data set of 182 proteins from the genome of 29 cyanobacteria with habitats in thermal springs, using the Allophycocyanin (APC) sequence from *Thermosynechococcus elongatus* (PDB code: 2V8A) as a query (Figure 1 and Table S1). We estimated an unrooted phylogenetic tree of all 182 hits, which provided the same relations among PBPs observed in the seminal work of Apt et al. (Figure S1).^30^ In detail, our study showed the presence of four main clades (Figure S1), composed mainly of Allophycocyanin subunit α (APC-α) with a population of 70 members, Allophycocyanins subunit β (APC-β) with 47, Phycocyanin α (PC-α) with 25, and Phycocyanin β (PC-β) with 31.^31,32^ The APC-α clade was found to contain proteins with over 31% early amino acids (Pro, Ala, Glu, and Gly), which strongly suggests its ancestral nature.^33^ To further investigate sequence relationships, and accounting for potential horizontal gene transfer, a clustering analysis was conducted (Figure S1).^34^ This analysis grouped the APC-α proteins into distinct clusters, further supporting the ancestral character of this clade. In addition, the estimation of a maximum likelihood phylogenetic tree indicated that the APC-α clade was the closest to the likely last universal common ancestor (LUCA) in our data set (Figure 1).^29^ Based on our findings, we considered the APC-α group should contain the most robust PBPs in terms of versatility and thermostability, *vide infra*.^24,35^ In particular, member 181 (APC-α from Synechococcus sp. OH28 taxonomy ID: 139350, here referred to as SyAPC-α) could be considered as the most versatile zombie-like PBP (i.e., proteins with a high ancestral character present in a living organism also referred to as a living fossil).^20^ This is related to (*i*) the shortest stepwise distance from the LUCA in comparison to the others (Figure 1) and (*ii*) its high ratio of ancestral amino acids (31.7%; sequence S1). Due to its zombie character and its hyperthermophilic organism of origin that keeps its fluorescence up to 80 °C,^32^ SyAPC-α was selected as a starting point to study the feasibility of PBPs to design visible emissive PBP variants for lighting applications.

**Figure 1:**
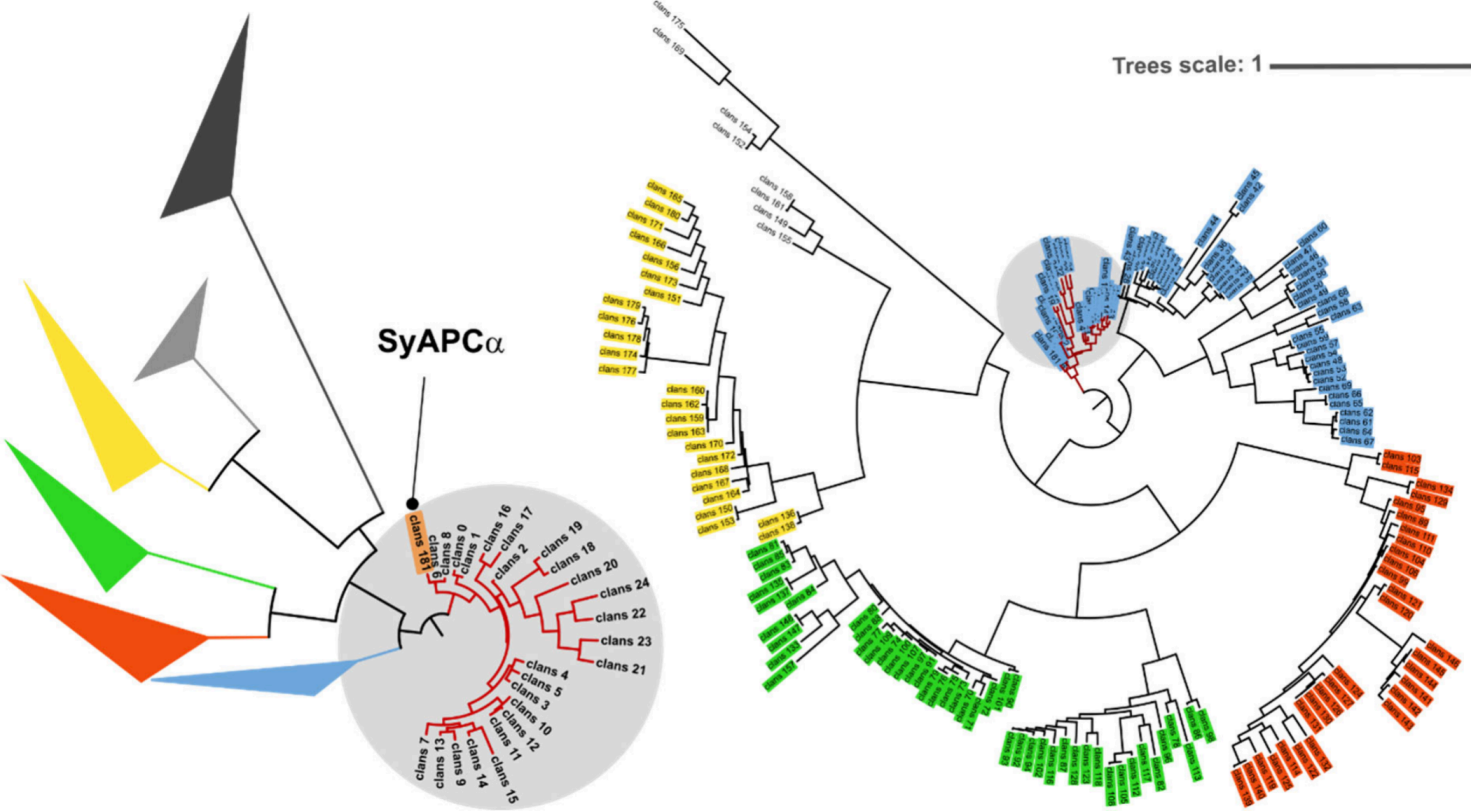
Maximum likelihood in an extended (right) and collapsed (left) phylogenetic tree, specifying four main branches corresponding to allophycocyanin subunit α (APC-α) in blue, allophycocyanin subunit β (APC-β) in green, phycocyanin subunit α (PC-α) in red, and phycocyanin subunit β (PC-β) in yellow. For improved visibility, the left tree indicates the living fossil in bold and collapsed branches for all branches. Gray circles highlight corresponding branches in extended (right) and collapsed (left) trees.

Among the production strategies of chromophorylated SyAPC-α in *E. coli*, including co-transformation with multiple plasmids to express the required enzymes for chromophore biosynthesis and attachment,^36^ or using a single plasmid encoding the complete metabolic pathway,^22,37^ we chose the latter in order to minimize the metabolic burden on *E. coli*.^38^ In this sense, we designed three plasmids expressing SyAPC-α and the metabolic pathway required to obtain the native deep-red-emitting PCB, the orange-emitting PEB, and the green-emitting PUB (Figure S2). Here, the plasmid for (phycoviolobilin) PVB was not designed, since this protein requires a more complicated metabolic route and the emission color will be in the same emission range as that of PEB.^38b^

Our constructs were integrated in a pET vector bearing a medium-strength origin of replication to maximize the cells’ growth rate and protein production by decreasing the metabolic load of the plasmid.^39^ Upon transformation in *E. coli* and induction, the PCB and PEB cells showed the expected marked blue and magenta color (Figure S2), confirming the expected promiscuity of the zombie-like PBP. Unfortunately, the PUB cells did not show any color, likely caused by insufficient expression or activity of the enzymes responsible for PUB synthesis. Future work will focus on optimizing the heterologous expression system in *E. coli*, even potentially transitioning to a fully *in vitro* approach, as a step toward achieving successful PUB chromophorylation.

Upon purification, SyAPC-α chromophorylated with PCB exhibited the expected deep-red emission centered at 640 nm (Figure S3), associated with emission lifetime (τ) values of 1.68 ns and a ϕ of 35%. SyAPC-α with PEB showed a bright orange emission at 564 nm (Figure 2A) with a τ and ϕ of 2.9 ns and 44%, respectively, associated with an extinction coefficient (ε) of 135 000 M^–1^cm^–1^. To confirm the thermostability of both proteins, we monitored the above figures upon thermal challenge at 75 and 90 °C over the course of 5 min. Surprisingly, the highest thermo-resilience was found for SyAPC-α with PEB, which kept its color to the naked eye and slightly decreased its ϕ to 40% after thermal challenge at 90 °C. In contrast, SyAPC-α with PCB lost a third of its ϕ at 75 °C and was fully denatured at 90 °C. Given the remarkable ability of the thermophilic-derived SyAPC-α to bind the non-native chromophore PEB, leading to exceptional thermo-resilience and bright orange emission, the SyAPC-α protein in complex with PEB was referred to as Synechococcus phycobiliprotein thermo-resilient zombie, or SPritZ. This representative was further tested in BioHLEDs *vide infra*.

**Figure 2:**
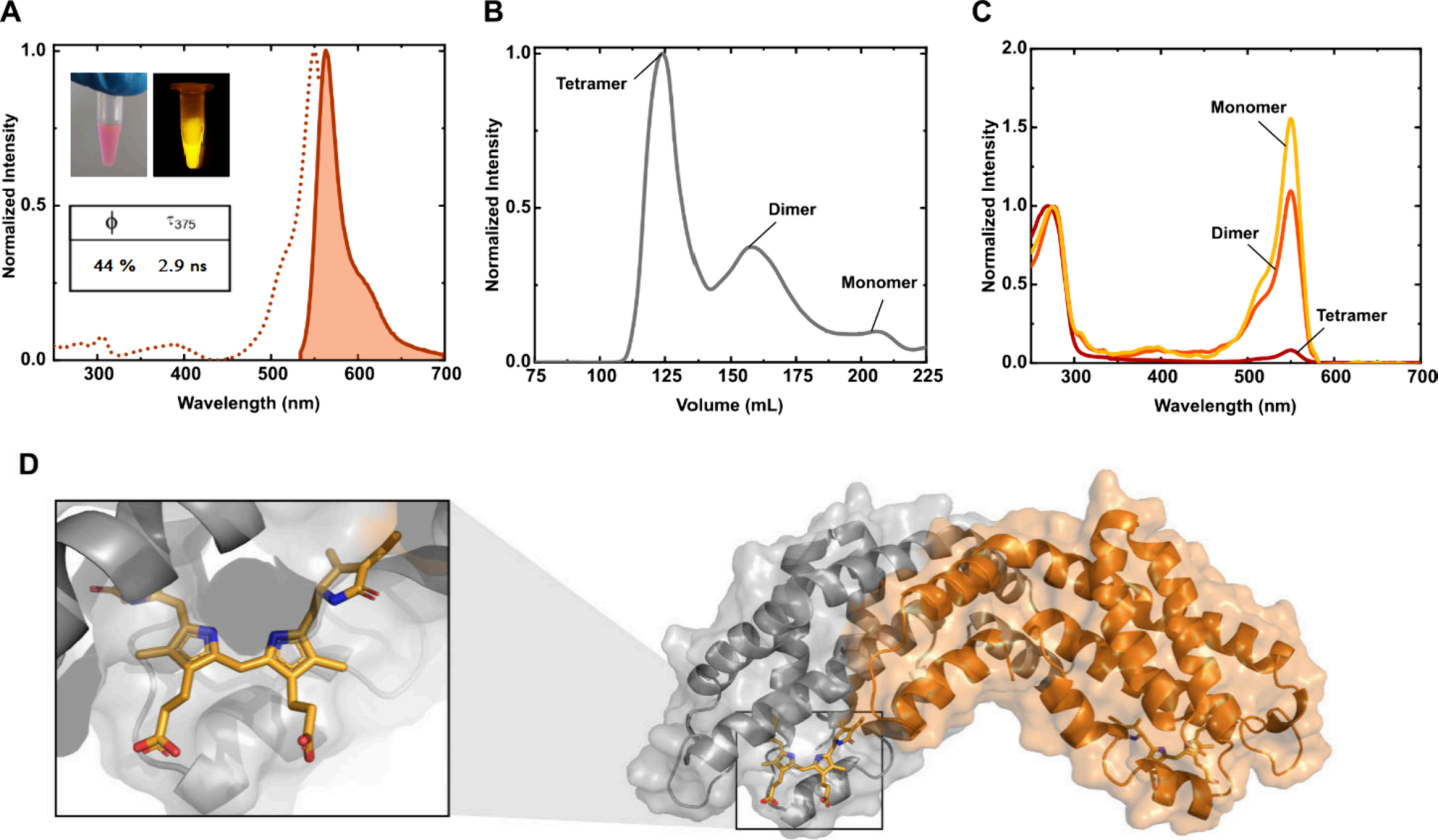
(A) Excitation (dotted line; λ_em_= 560 nm) and emission (solid line; λ_exc_= 530 nm) spectra of SPritZ in PBS aqueous solution. Inset: SPritZ under ambient (left) and blue light (right) illumination. (B) Size exclusion chromatography (SEC) of SPritZ. The data were collected after an affinity chromatography purification step. (C) Absorption spectra corresponding to each SEC fraction of SPritZ in PBS aqueous solution. (D) AlphaFold2 model of homodimeric SPritZ chromophorylated with PEB. The chromophore was added using Coot, while the structure was relaxed with Rosetta (see Supporting Information).

Next, we carefully analyzed the purification process of SPritZ via affinity and size exclusion chromatography, encountering three main peaks: a homotetrameric structure (Figure 2B) with a minimal chromophorylation as indicated by the absorption spectra (Figure 2C) and the homodimer and monomer peaks with a high degree of chromophorylation (Figure 2C). AlphaFold2 ^40^ predictions suggest that the tetramer assembly causes steric hindrance at the chromophore binding site (modeled in Figure S4), possibly hampering the activity of the lyase CpcS. Concerning the monomer and homodimer protein production, the majority of the chromophorylated protein existed in a homodimer conformation (modeled via AlphaFold2 in Figure 2D), but the 280/550 nm absorption intensity ratio indicates that chromophorylation is more effective in the monomer (Figure 2C). Thus, the homodimer SPritZ proteins should consist of a mixture of fully and partially chromophorylated homodimers (i.e., those with only one PEB per dimeric unit). This finding could be in line with the typical behavior of engineered homo-oligomeric PBPs, such as smURFP, which tend to exhibit a single chromophore despite their dimeric conformation.^41^ The monochromophorylation of smURFP is dependent on its oligomerization, where a bound chromophore stabilizes the dimeric interface, but simultaneously precludes the binding of a second chromophore to the protein backbone.^42^ However, the chromophore cavity of SPritZ has not been optimized to efficiently host the non-native PEB chromophore, and this could affect a full chromophorylation. Indeed, phycoerythrins (PEs) that naturally bind PEB present a lysine (K) immediately adjacent to the cysteine (C) responsible for the covalent bond with PEB (C81).^43^ This residue (K80) assists in establishing electrostatic/H-bonding interactions with the carboxyl-terminal on the C-ring of PEB, increasing the rigidity of the system, which results in enhanced chromophore stability. A further corroboration came from the analysis of the AlphaFold2 model of SPritZ harboring the desired mutation (T80K) and the chromophore PEB. Our relaxed model indicated the creation of a salt bridge between K80 and the B-ring carboxyl-terminal of PEB in the SPritZ variant (Figure 3A). While this model does not fully account for solvent interactions, it could be possible that stabilization also occurs through a H-bonding interaction, as observed in other PEs.^43^

**Figure 3:**
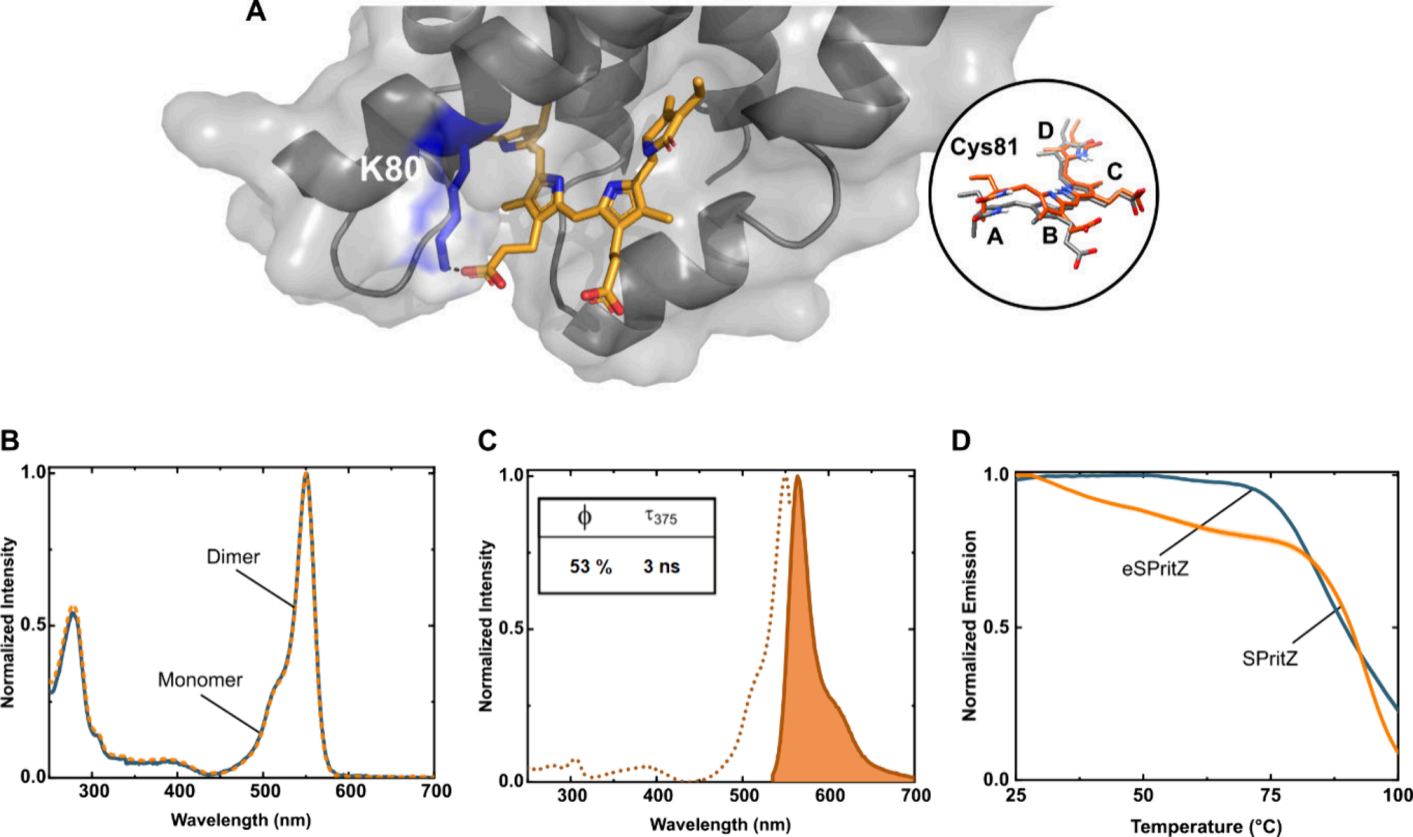
(A) Model of eSPritZ obtained with AlphaFold2 and relaxed via Rosetta (see Supporting Information). PEB is indicated in orange, and K80 is indicated in blue. The inset shows the superposition of PEB found in SPritZ (gray) and eSPritZ (orange). (B) Absorption spectra of eSPritZ monomers and homodimers in PBS aqueous solution. (C) Excitation (dotted line; λ_em_= 570 nm) and emission (solid line; λ_exc_= 540 nm) spectra of eSPritZ in PBS aqueous solution. (D) *T*_nr_ of SPritZ and eSPritZ in PBS buffer solutions.

In light of these findings, we set out to produce, purify, and characterize this variant. In contrast to SPritZ, the T80K variant nicely showed a higher degree of chromophorylation, since the absorption intensity and the 280/550 intensity ratio of the monomeric and homodimeric species present almost identical spectra (Figure 3B). What is more, the T80K variant featured the same emission and excitation spectra as those of SPritZ (Figures 2A and 3C), but a ca. 10% increase in ϕ, reaching values of 53% associated with longer τ values of 3 ns. Along this line, the ε of 153 000 M^–1^ cm^–1^ also increased, resulting in an enhanced brightness (59 vs 81). Finally, the positive effects of T80K are also reflected in the refolding temperature measured by modulated scanning fluorimetry (MSF).^44^ Here, the emission intensity of the T80K variant holds until ca. 75 °C, resulting in a temperature of nonreversibility (*T*_nr_) area increase from 57 to 63 (Figure 3D), confirming the superior refolding capability of the T80K variant. This confirms the remarkable thermal resilience of a protein evolved in a thermophilic organism. Overall, owing to the outperforming chromophorylation, photoluminescence, and thermal resilience, the T80K variant is hereafter referred to as enhanced SPritZ (eSPritZ).

Next, SPritZ and eSPritZ were integrated in polymer coatings using a fully biogenic matrix made of hydroxypropyl cellulose (HPC) following the published procedure (see experimental section).^23^ This was already proven to be the most effective matrix to preserve the native features of phycobiliproteins in waterless dome-shape coatings suitable for photon down-conversion in lighting applications (Figure 4A).^21^ The photophysical characterization of both self-standing coatings harboring up to 0.75 mg of each protein consists of similar excitation and emission spectra slightly broadened compared to those in solution due to protein aggregation and/or chromophore distortion upon interaction with HPC (Figures 4B and S5). In detail, the ϕ and τ values remain in the range of 48% and 3 ns for eSPritZ-HPC coatings, while their thermal behavior is better than that in PBS buffer solution, showing a similar *T*_nr_ (Figure S6), but an enhanced *T*_m_ (measured at 50% initial intensity) going from 56 to 77 °C (Figure 4C). In comparison, SPritZ-HPC coatings showed similar photoluminescence figures but worse thermal features (Figures S5 and 4C).

**Figure 4:**
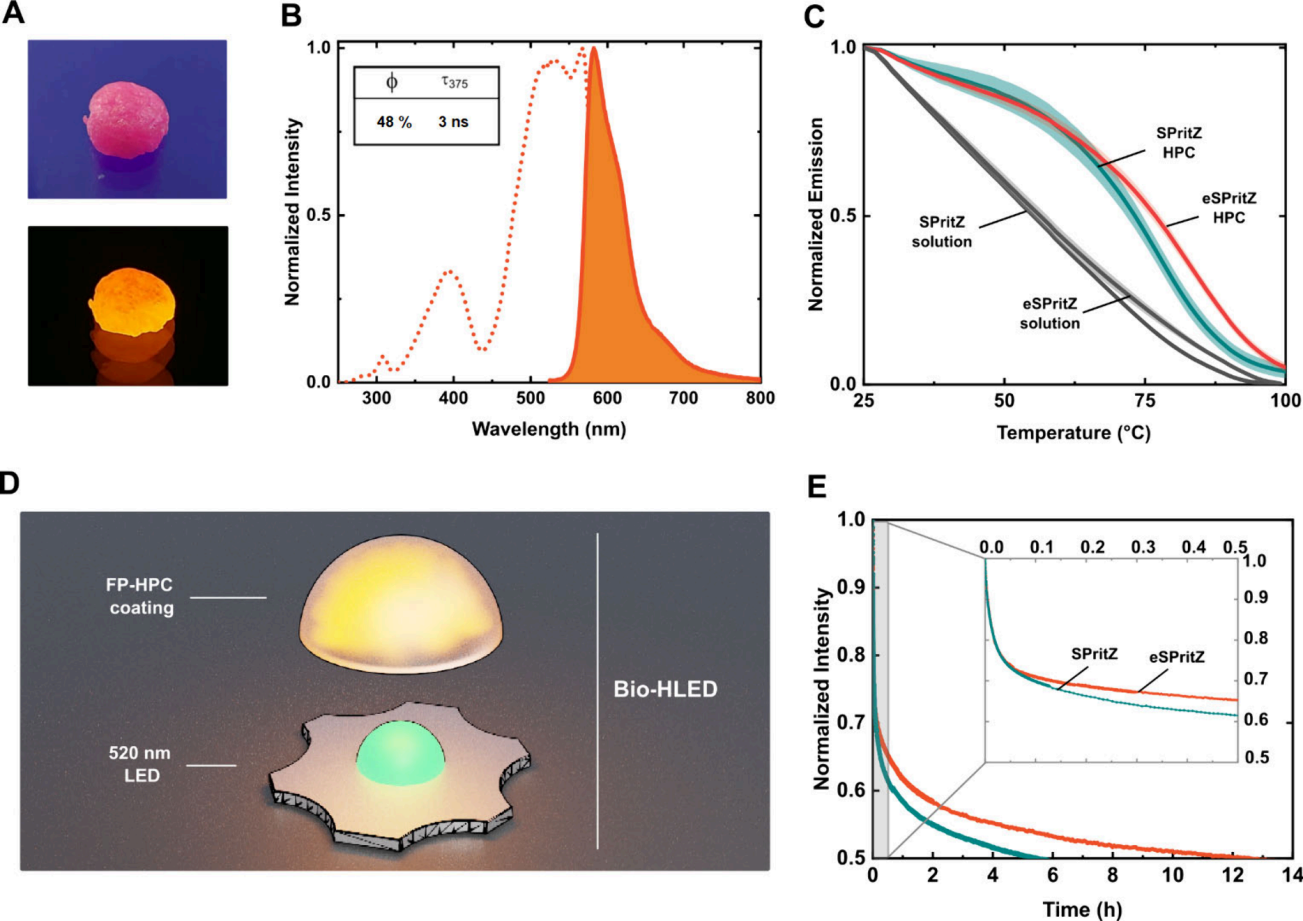
(A) Pictures of eSPritZ-HPC color down-converting coatings under ambient (top) and blue (bottom) illumination. (B) Excitation and emission spectra of eSPritZ-HPC coatings. Excitation (dotted line; λ_em_ = 580 nm) and emission (solid line; λ_exc_ = 565 nm) spectra of the protein coatings. (C) *T*_m_ of SPritZ and eSPritZ in either PBS aqueous solution or embedded in HPC. (D) BioHLED schematic overview. (E) Photostability of devices prepared with SPritZ and eSPritZ-HPC coatings operating at 200 mA. Inset: zoom-in on the first 30 min.

The above coatings were easily integrated as color filters in the BioHLED devices (Figure 4D). They consisted of a commercial green-emitting pumping LED (520 nm) that is directly covered with the above dome-shaped protein–polymer coating. These devices were driven at high applied currents of 200 mA (90 mW/cm^2^), and the emission spectra were monitored over time (Figures 4E and S7). In eSPritZ devices, the initial spectra resemble that of the photoluminescence features of the coatings with an almost quantitative LED emission conversion (<5% remaining LED intensity; Figure S7) that results in an overall initial orange emission with *x/y* CIE color coordinates of 0.59/0.39 and a color purity of 0.95. The same protein amount of SPritZ led to a lower LED conversion with a prominent remaining green emission that leads to color corruption (yellowish orange color with *x/y* CIE coordinates of 0.53/0.44; Figure S7). This is expected, as the eSPritZ shows an enhanced brightness (59 vs. 81; *vide supra*) compared to SPritZ, enabling us to use less protein to meet the desired LED conversion and color targets. Besides this, the same amount of protein allowed us to carry out a fair device stability comparison between both proteins.

In detail, the protein emission intensity decreased following a two-step process for both protein devices (Figure 4E). At first, a similar first exponential decay was noted, reaching up to ca. 30% loss of the initial intensity during the first 5 min (Figure 4E, inset). This sudden decrease in emission has been typically attributed to emission thermal quenching as the device temperature reaches values of 60–80 °C.^8–16^ However, the device working temperature rises up to 35 ± 2 °C for both devices (Figure S8). In addition, the emission intensity related to the LED (520 nm) does not increase over this time (Figure S7), indicating that the early photobleaching of proteins that are insufficiently stabilized in the HPC matrix could be ruled out. Thus, this initial exponential decay could be tentatively attributed to a combination of thermal emission quenching, irreversible photoconversion of PEB into nonemissive, yet absorbing, species, and/or the conformational changes of the chromophore that promote the nonradiative deactivation via dark states as reported in solution.^45^ These hypotheses should be corroborated in HPC coatings by, for example, transient absorption and emission spectroscopy assays and/or temperature-dependent spectroscopy. In the second step, the protein emission intensity decays in a linear fashion with a steeper slope for SPritZ, reaching an overall device half-life (*t*_50_) average of 5 h for SPritZ and 12.5 h for eSPritZ (Figure 4E). During this regime, the LED emission intensity increases correspondingly, suggesting that both proteins are slowly photobleached via, for example, photon-induced oxidation.^9,12,23,45^ Overall, these findings suggest that the T80K mutation plays a crucial role both in the chromophorylation (*vide supra*) and in reducing its photobleaching rate by a 2.5-fold factor in polymer coatings.

In light of the above discussion, this work positively answers the question of whether FPs present in extremophile organisms and/or living fossils will be of high interest for lighting purposes. Among them, we focused on those with the light-harvesting protein system based on PBPs that have been proposed to replace classical β-barrel FPs as fluorescent labels in cytometry and immunofluorescence analysis but not yet explored for lighting purposes. More specifically, we have disclosed a comprehensive phylogeny of PBPs using a large data set (182 proteins present in 29 thermophilic organisms) to identify a PBP chosen for its “living fossil” character and its ability to bind the non-native chromophore PEB, producing a bright and thermal-stable orange-emitting variant. This FP, initially referred to as SPritZ, was engineered to harbor a single mutation near the chromophore binding site (T80K), which enhanced chromophorylation, ϕ, and thermal resilience, resulting in an enhanced variant coined as eSPritZ. Both SPritZ and eSPritZ showed an excellent compatibility with HPC matrices and exhibited high thermo-resilience, while eSPritZ demonstrated substantial improvements in BioHLED stability (2.5-fold increase) compared to those with SPritZ. These findings successfully establish a proof-of-concept for the integration of another class of thermophilic-derived FPs in lighting devices, offering efficient, visible-range emission for sustainable photonics and optoelectronics. However, device lifetime must be improved to reach device stabilities like those noted with classical β-barrel FPs. Ongoing work on protein design of SPritZ and other zombie-like PBPs (Figure 1) as well as the formation of high-order oligomers as noted in Nature is the focus of our current research.

## Supplementary Material



## Notes

Materials and methods; supporting results for unrooted phylogenetic tree; schematic of protein expression vectors; excitation/emission spectra of SyAPC-α chromophorylated with PCB; predicted model of tetrameric SPritZ; excitation/emission spectra of SPritZ-HPC coating; *T*_nr_ of SPritZ and eSPritZ in HPC coatings; spectra of devices implementing SPritZ and eSPritZ; list of thermophilic cyanobacteria; SyAPC-α amino acid sequence (PDF)

## ABBREVIATIONS

FP: fluorescent protein
PBP: phycobiliprotein
PEB: phycoerythrobilin
PCB: phycocyanobilin
PUB: phycourobilin
SyAPC-α: Synechococcus allophycocyanin α
SPritZ: Synechococcus phycobiliprotein thermo-resilient zombie
eSPritZ: enhanced Synechococcus phycobiliprotein thermo-resilient zombie
MSF: modulated scanning fluorimetry
HPC: hydroxypropyl cellulose
BioHLED: biohybrid light-emitting diode
ϕ: photoluminescence quantum yield
τ: lifetime
ε: extinction coefficient.
