# Supplementary material for: Bright Thermo-resilient and Promiscuous Zombie Protein for Lighting Applications
Source: ACS Mater Lett. 2025 Jul 25;7(9):3041–8. doi: 10.1021/acsmaterialslett.5c00653 (PMC12406245; doi:10.1021/acsmaterialslett.5c00653)
Supplement: Supplementary file 1 [file tz5c00653_si_001.pdf]

Supplementary Information

Bright Thermo-resilient and Promiscuous Zombie Protein for Lighting Applications

Marta Patrian, Marco Hasler, Jesús A. Banda-Vázquez, Evgenia Borisova, Juan Pablo Fuenzalida Werner, and Rubén D. Costa*

Technical University of Munich, Campus Straubing for Sustainability and Biotechnology, Chair of Biogenic Functional Materials, Schulgasse, 22, Straubing 94315, Germany. E-mail: ruben.costa@tum.de

Table of Content

Materials and methods **3**
Sequence alignment, phylogenetic estimation and clustering analysis 3
Protein production 3
Computational characterization techniques 3
Experimental characterization techniques 3
Modulated scanning fluorimetry 4
Device fabrication and characterization 4
Figures and tables **5**
Figure S1 5
Figure S2 6
Figure S3 6
Figure S4 7
Figure S5 7
Figure S6 7
Figure S7 8
Figure S8 8
Table S1. 9
Sequence 10
References 11

Materials and methods

Sequence alignment, phylogenetic estimation and clustering analysis

The multiple sequence alignment of the data set was done with MAFFT [1] and the Phylogenetic tree was estimated with PhyML online[2] using defaults parameters. The clustering analysis was done using CLANS on the same data set.[3] The tree was visualized using iTOL online.[4]

Protein production

All fluorescent proteins were produced in *Escherichia coli* BL21 (DE3) cells in liquid lysogeny broth (LB). The main cultures were induced with Isopropyl-β-D-1-thiogalactopyranoside when they reached OD_{600} 0.6 and grown for 48 h at 16 °C. Cells were harvested by centrifugation at 5,000 g for 30 min, washed with PBS, and then the pellet was disrupted *via* sonication (80 amplitude, 1 s on, 3 s off). The sonicated cells were centrifuged to separate soluble fraction and cell debris at 13,500 g. The His-tagged proteins in the supernatants were purified using a Ni^{2+} His-Trap column and desalted. Size-exclusion chromatography was carried out for SPritZ and eSPritZ to obtain the fractions of tetramer, dimer, and monomer. The proteins were flash-frozen in PBS buffer and stored at −80 °C. Before use, the proteins were thawed and centrifuged to remove aggregated proteins.

Computational characterization techniques

SPritZ and eSPritZ homo-dimers and homo-tetramers were modelled using AlphaFold2.[5] The highest ranking model was selected and relaxed 1,000 times with tools within the Rosetta suite.[6,7] Chromophore were added to the structures using the protein modelling software Coot[8a] and visualized using PyMol.[8b]

Preparation of protein matrices

The HPC coatings were prepared by dissolving in type I water HPC, with a concentration of 230 mg mL^{-1}. Then, the HPC solution was mixed with the FP solution. The coatings were obtained after stepwise drying of the solution by vacuum in a dome-shaped mold. Coatings were stored at room temperature.

Experimental characterization techniques

Absorption spectra of thawed FP solutions were recorded with a UV–vis-2600i spectrophotometer (Shimadzu) from 250 to 900 nm, with slow scan speed, a data interval of 1 nm, and 1 nm slid width. Extinction coefficient of PBPs was determined based on the comparison between the absorbance values of the Q peak and the absorbance values of the Soret peak, assuming the latter to have the same ε of the free tetrapyrrole, which is set at 18,300 $M^{-1}cm^{-1}$ for PEB and 32,600 $M^{-1}cm^{-1}$ for PCB. The following formula was applied: $\text{Q band} = \frac{\text{Abs max Q band}}{\text{Abs max Soret band}} \times \varepsilon \text{ Soret band}$.

Photophysical studies were carried out at room temperature using an FS5 spectrofluorometer (Edinburgh Instruments) with a SC-10 module for solid samples, SC-05 for liquid samples. A 375 nm time-correlated single photon-counting module was used to determine τ and adjusted to an exponential decay fit with Origin 2021b (OriginLab Corporation, Northampton, MA, USA).

The average τ was calculated based on the following formula: $< \tau > = \frac{\int_0^x t \sum a_i \, exp\left(-\frac{t}{\tau_i}\right)dt}{\int_0^x \sum a_i \, exp\left(-\frac{t}{\tau_i}\right)dt} = \frac{\sum a_i \tau_i^2}{\sum a_i \tau_i}$; where a_i (λ) is the amplitude fractions and τ_i are the lifetimes. ϕ was determined using a Quantaurus-QY Absolute PL quantum yield spectrometer C11347-11 (Hamamatsu Photonics).

Modulated scanning fluorimetry

Modulated Scanning Fluorimetry was performed as previously described. [9] The Thermocycler CFX96 Touch Real-time PCR System (Bio-Rad) was employed to perform MSF measurements. One program composed of heating and cooling cycles ranging from 25 to 99 °C was used to measure the progressive loss of fluorescence and the irreversible unfolding of the FPs studied in this work. The samples were heated at 5 °C s^{-1} and held for 1 min at the temperature peak, followed by a recovery period of 5 min at 25 °C. The thermograms were buffer-subtracted and normalized by the highest fluorescence intensity of each sample. Data analysis was performed using Origin 2021b (OriginLab Corporation, Northampton, MA, USA). Mean values and standard deviations of quintuplicates were calculated and plotted. Modulated scanning fluorimetry curves were obtained by plotting the fluorescence values obtained at 25 °C.

Device fabrication and characterization

The dome-shaped phosphors (*ca*. 10, 4, and 2 mm for diameter, height, and thickness) were placed directly on top of the unmodified 520 nm LED (1 W, WINGER Electronics), as referred as on-chip in previous works. The power of the LEDs were measured with a PM100D device with a S121C detector from THORLABS. This was driven at 200 mA at ambient conditions. Duplicates of devices prepared from two different protein productions were compared with a deviation of 17 % and 10 % for SpritZ and eSpritz, respectively. To operate the LED a Keithley 2231A-30-3 was used. Electroluminescence spectra were recorded with an AvaSpec-ULS2048CL-EVO spectrophotometer and an AvaSphere-30 integrating sphere. The temperature was monitored using a thermographic camera ETS320 (FLIR).

Figures and tables

Figure S1

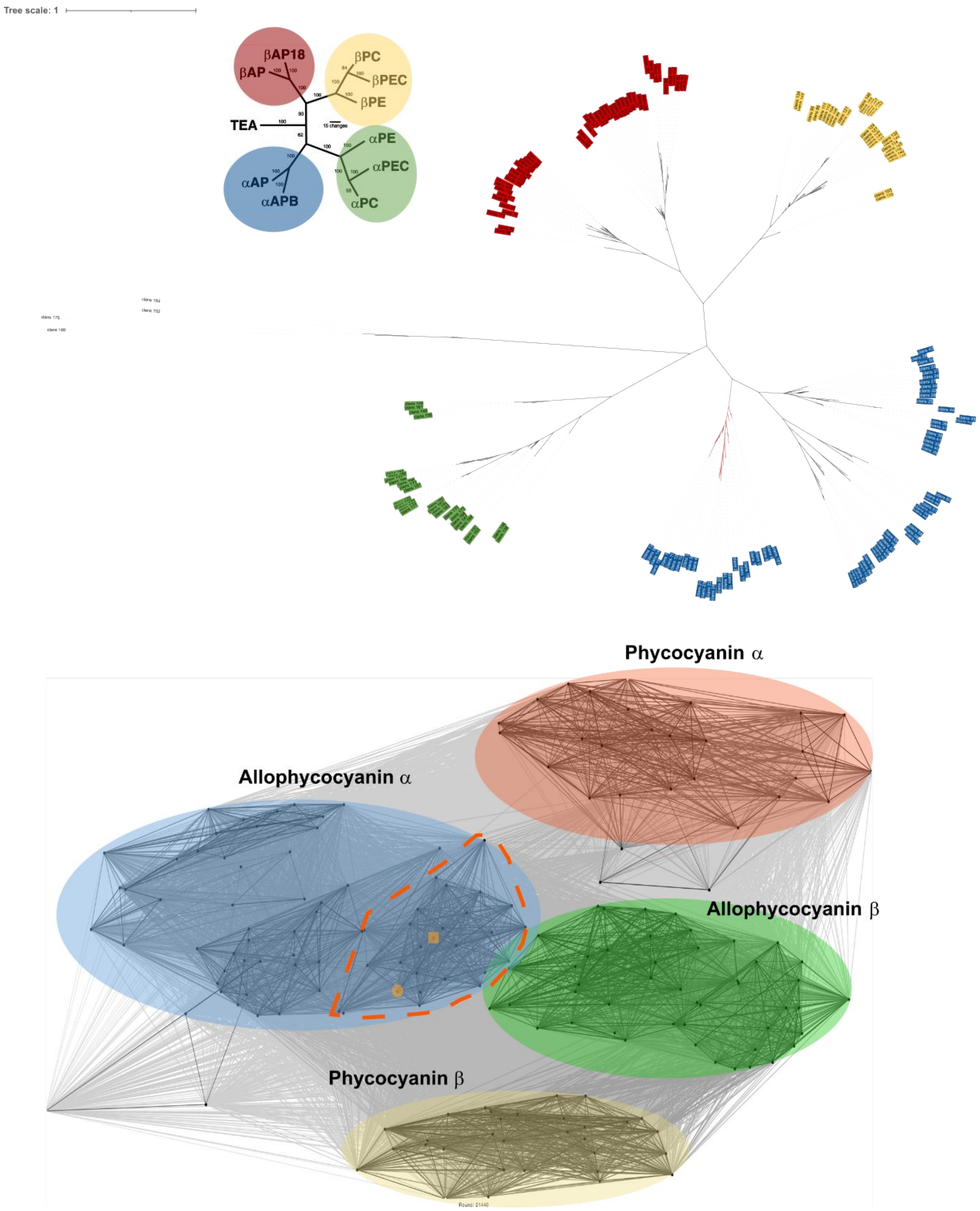

Figure S1. Top: unrooted phylogenetic tree of 182 candidates created in this work (right) and previously determined by Apt *et al.* (inset, left). Bottom, Similarity network composed of four main clusters: Allophycocyanin subunit alfa (APC-α, in blue), Allophycocyanins subunit beta (APC-β, in green), Phycocyanin alpha (PC-α, in red), and Phycocyanin beta (PC-β, in yellow).

Figure S2

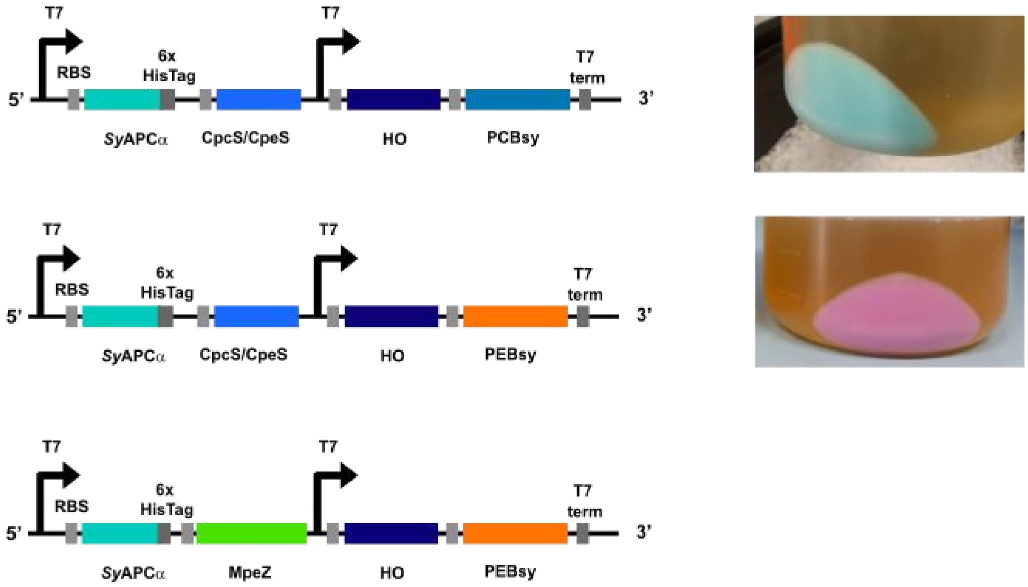

Figure S2. Schematic of the expression vector used for the production of *Sy*APC-α chromophorylated with PCB (top), PEB (middle), and PUB (bottom).

Figure S3

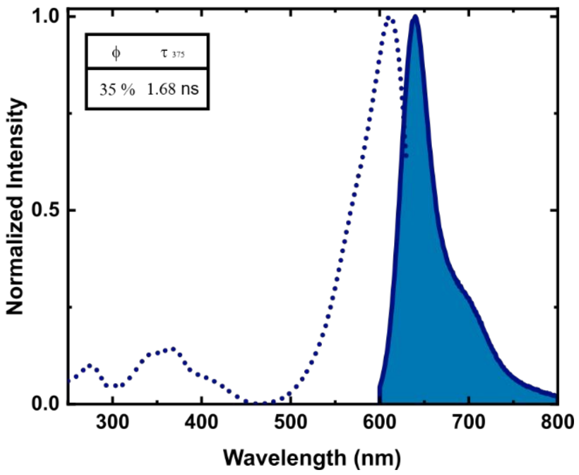

Figure S3. Excitation (dotted line; λ_{em} = 640 nm) and emission (solid line; λ_{exc} = 590 nm) spectra of *Sy*APC-α chromophorylated with PCB.

Figure S4

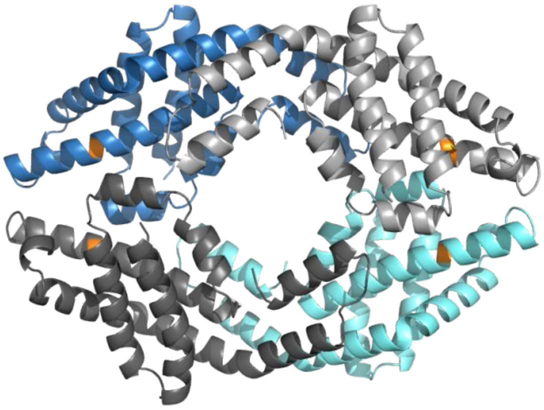

Figure S4. AlphaFold2 model of tetrameric SPritZ. The four subunits are indicated in different colors. C81 is indicated in orange on each subunit.

Figure S5

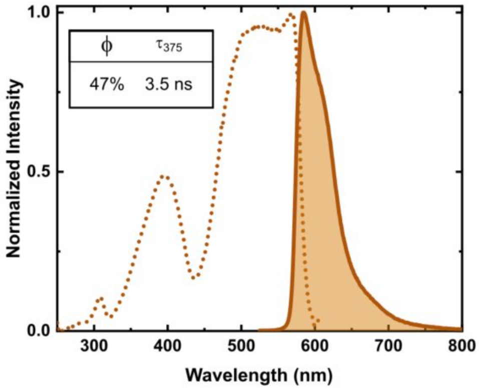

Figure S5. Excitation and emission spectra of SPritZ-HPC coating.

Figure S6.

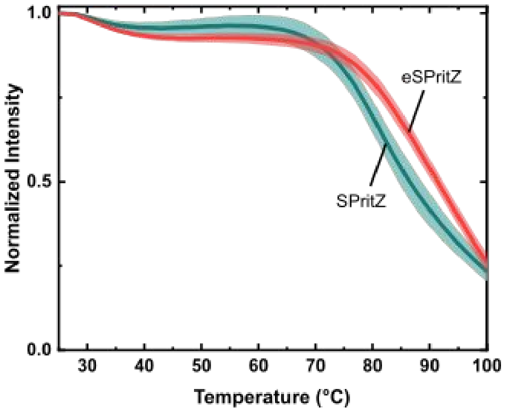

Figure S6. T_{nr} of SPritZ-HPC and eSPritZ-HPC coatings.

Figure S7

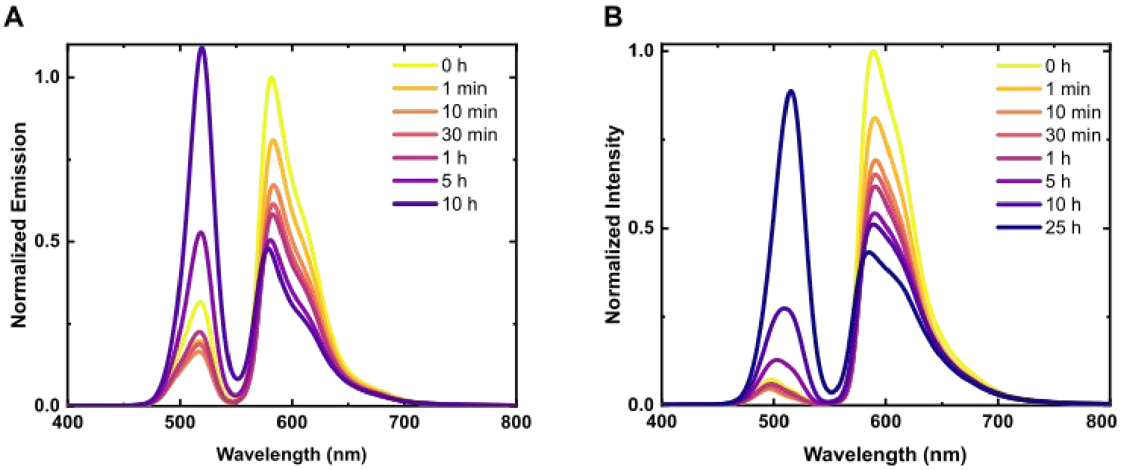

Figure S7. Emission spectra over time of devices with SPritZ-HPC (A, C) and eSPritZ-HPC (B, D) coatings operating at 200 mA.

Figure S8

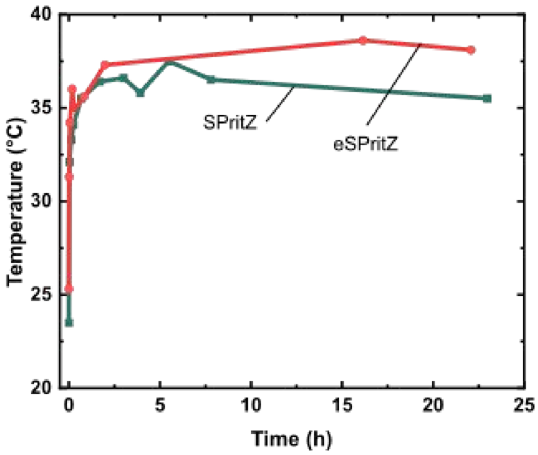

Figure S8. Working temperature of devices operating at 200 mA and implementing SPritZ-HPC and eSPritZ-HPC.

Table S1.

Table S1. Cyanobacteria with habitats in thermal spring strain name and taxa id.

Strain Name	Isolated From	NCBI:txid
Nostoc_sp._FACHB-857_cyanobac	Hot Spring	2692840
Chlorogloeopsis_fritschii_PCC-9212_GCA_000317265.1	Hot Spring	184925
Chroogloeocystis_siderophila_5.2_s.c.1_GCA_001904655.1	Hot Spring	247279
Cyanobacterium_aponinum_B-1201_GCA_002736005.1	Hot Spring	2047986
Cyanobacteria_bacterium_PCC-7702_GCA_000332255.1	Hot Spring	1173021
Fischerella_major_NIES-592_GCA_001904645.1	Hot Spring	210994
Fischerella_muscicola_PCC-7414_GCA_000317205.1	Hot Spring	306281
Fischerella_sp._NIES-3754_GCA_001548455.1	Hot Spring	1752063
Fischerella_thermalis_PCC-7521_GCA_000317225.1	Hot Spring	98439
Gloeocapsa_sp._PCC-7428_GCA_000317555.1	Hot Spring	1173026
Hydrococcus_rivularis_NIES-593_GCA_001904635.1	Hot Spring	1921803
Leptolyngbya_sp_JSC-1_GCA_000733415.1	Hot Spring	1487953
Leptolyngbya_sp_O-77_GCA_001548395.1	Hot Spring	1080068
Mastigocladus_laminosus_UU774_GCA_000934435.1	Hot Spring	1594576
Nostoc_sp_PCC-7524_GCA_000316645.1	Hot Spring	28072
Cyanobacteria_bacterium_MTP1_GCA_001482745.1	Hot Spring	1751289
Pleurocapsa_sp_PCC-7327_GCA_000317025.1	Hot Spring	118163
Synechococcus_lividus_PCC-6715_GCA_002754935.1	Hot Spring	1917166
Synechococcus_sp._60AY4M2_GCA_002760375.1	Hot Spring	1353262
Synechococcus_sp._63AY4M1_GCA_002760395.1	Hot Spring	1353263
Synechococcus_sp._63AY4M2_GCA_002760475.1	Hot Spring	1353266
Synechococcus_sp._65AY640_GCA_002760445.1	Hot Spring	1353264
Synechococcus_sp._65AY6A5_GCA_002760415.1	Hot Spring	1353265
Synechococcus_sp._65AY6Li_GCA_002760345.1	Hot Spring	1351840
Synechococcus_sp._JA-2-3Ba2-13_GCA_000013225.1	Hot Spring	321332
Synechococcus_sp_JA-3-3Ab_GCA_000013205.1	Hot Spring	321327
Synechococcus_sp_OH28	Hot Spring	139350
Thermosynechococcus_elongatus_BP-1_GCA_000011345.1	Hot Spring	197221
Thermosynechococcus_sp_NK55a_GCA_000505665.1	Hot Spring	1394889

Sequence

>SyAPCα

MSVITKSIVNADAEARYLSPGELDRIKSFVASGEKRLRIAQVLTESRERIVKQAADQLFQKRPDIVSPGGNAYGEEMT
ATCLRDMDYYLRLITYGIVAGDVTPIEEIGLVGVREMYNSLGTPLPAVAESIRLMKQVAMGLLSPEDAAEAAYYFDFV
AGAMSEF

References

(1) Katoh, K.; Rozewicki, J.; Yamada, K. D. MAFFT Online Service: Multiple Sequence Alignment, Interactive Sequence Choice and Visualization. *Briefings in Bioinformatics* **2019**, *20*, 1160.

(2) Guindon, S.; Dufayard, J.-F.; Lefort, V.; Anisimova, M.; Hordijk, W.; Gascuel, O. New Algorithms and Methods to Estimate Maximum-Likelihood Phylogenies: Assessing the Performance of PhyML 3.0. *Systematic Biology* **2010**, *59*, 307.

(3) Frickey, T.; Lupas, A. CLANS: A Java Application for Visualizing Protein Families Based on Pairwise Similarity. *Bioinformatics* **2004**, *20*, 3702.

(4) Letunic, I.; Bork, P. Interactive Tree of Life (iTOL) v6: Recent Updates to the Phylogenetic Tree Display and Annotation Tool. *Nucleic Acids Research* **2024**, *52*, W78.

(5) Jumper, J.; Evans, R.; Pritzel, A.; Green, T.; Figurnov, M.; Ronneberger, O.; Tunyasuvunakool, K.; Bates, R.; Žídek, A.; Potapenko, A.; Bridgland, A.; Meyer, C.; Kohl, S. A. A.; Ballard, A. J.; Cowie, A.; Romera-Paredes, B.; Nikolov, S.; Jain, R.; Adler, J.; Back, T.; Petersen, S.; Reiman, D.; Clancy, E.; Zielinski, M.; Steinegger, M.; Pacholska, M.; Berghammer, T.; Bodenstein, S.; Silver, D.; Vinyals, O.; Senior, A. W.; Kavukcuoglu, K.; Kohli, P.; Hassabis, D. Highly Accurate Protein Structure Prediction with AlphaFold. *Nature* **2021**, *596*, 583.

(6) Nivón, L. G.; Moretti, R.; Baker, D. A Pareto-Optimal Refinement Method for Protein Design Scaffolds. *PLOS ONE* **2013**, *8*, e59004.

(7) Alford, R. F.; Leaver-Fay, A.; Jeliazkov, J. R.; O'Meara, M. J.; DiMaio, F. P.; Park, H.; Shapovalov, M. V.; Renfrew, P. D.; Mulligan, V. K.; Kappel, K.; Labonte, J. W.; Pacella, M. S.; Bonneau, R.; Bradley, P.; Dunbrack, R. L. Jr.; Das, R.; Baker, D.; Kuhlman, B.; Kortemme, T.; Gray, J. J. The Rosetta All-Atom Energy Function for Macromolecular Modeling and Design. *J. Chem. Theory Comput.* **2017**, *13*, 3031.

(8) a) Emsley P.; Lohkamp, B; Scott, W. G.; Cowtan, K. Features and development of Coot. *Acta Crystallogr D Biol Crystallogr.* **2010**, *66*, 486. b) The PyMOL Molecular Graphics System, Version 3.0 Schrödinger, LLC. https://www.pymol.org

(9) Svilenov, H. L.; Menzen, T.; Richter, K.; Winter, G. Modulated Scanning Fluorimetry Can Quickly Assess Thermal Protein Unfolding Reversibility in Microvolume Samples. *Mol Pharm* **2020**, *17*, 2638.
